# Correlation between antibutyrylcholinesterasic and antioxidant activities of three aqueous extracts from Tunisian *Rhus pentaphyllum*

**DOI:** 10.1186/1476-0711-10-32

**Published:** 2011-08-31

**Authors:** Hedi Ben Mansour, Sonia Yatouji, Sihem Mbarek, Ikram Houas, Afef Delai, Dorra Dridi

**Affiliations:** 1 Institut Supérieur de Biotechnologie (ISB), Technopole Sidi Thabet, Université la Manouba 2020 Ariana Tunisie; 2 Unité 05/UR/09-09, Mécanismes Moléculaires et Pathologies, Faculté de Médecine de Monastir, 5019 Monastir, Tunisie

**Keywords:** *Rhus pentaphyllum*, anti-Butyrylcholinesterasic activity, free radical scavenging activity, antioxidant activity

## Abstract

For centuries, plants have been used in traditional medicines and there has been recent interest in the chemopreventive properties of compounds derived from plants. In the present study, we investigated the antibutyrylcholinestrasic (anti-BuChE) and antioxidant (against some free radicals) activities of extracts from *Rhus pentaphyllum*. Aqueous extracts were prepared from powdered *R. pentaphyllum *roots, leaves and seeds and characterized for the presence of tannins, flavonoids and coumarins. Seeds aqueous extract contained the highest quantities of both flavonoids and tannins (21.12% and 17.45% respectively). In the same way, seeds extracts displayed remarkable inhibition against BuChE over 95%, at 100 μg/ml and with IC_50 _0.74 μg/ml. In addition, compared to leaves and roots extracts, seeds aqueous extract revealed relatively strong antiradical activity towards the ABTS^**.+ **^(IC_50 _= 0.25 μg/ml) and DPPH (IC_50 _= 2.71 μg/ml) free radicals and decreased significantly the reactive oxygen species such O_2_^.- ^(IC_50 _= 2.9 μg/ml) formation evaluated by the non-enzymatic generating O_2_^.- ^system (Nitroblue tetrazolium/riboflavine). These data suggest that the anti-BuChE activities mechanism of these extracts occurs through a free radical scavenging capacities.

The present study indicates that extracts of *Rhus pentaphyllum *leaves, seeds and roots are a significant source of compounds, such as tannins, flavonoids and coumarins, with anti-BuChE and antioxidant activities, and thus may be useful for chemoprevention.

## Introduction

Alzheimer's disease (AD) is a degenerative neurological disorder characterized by senile plaques containing amyloid β protein and loss of cholinergic neuromediators in the brain [1,2]. The most remarkable biochemical change in AD patients is a reduction of acetylcholine (ACh) levels in the hippocampus and cortex of the brain [3]. Therefore, inhibition of acetylcholinesterase (AChE), the enzyme responsible for hydrolysis of ACh at the cholinergic synapse, is currently the most established approach to treating AD [4]. While AChE is found in all excitable tissue, whether nerve or muscle, in most erythrocytes and in placental tissue, BChE is present more commonly in the body including the central and peripheral nervous system, liver and plasma [5]. On the other hand, oxidative stress caused by reactive oxygen species (ROS), is known to cause the oxidation of biomolecules leading to cellular damage. It is also speculated to be pathologically important in various neurodegenerative processes including cognitive deficits that occur during normal cerebral aging, Alzheimer's disease (AD) and Parkinson's disease [6-8]. Nowadays, the most accepted theory about the disturbing effect of free radicals in the process of aging was reported by Harman [9]. Later on, it was also reported that oxidative stress is associated with the pathogenesis of AD and cellular characteristics of this disease are either causes or effects of oxidative stress [10,11].

These evidences clearly show that oxidative stress, an early event in AD, may play a key pathogenic role in the disease [12]. Interestingly, intake of polyphenols through diets rich in fruits, vegetables and beverages such as red wine was stated to reduce incidence of certain age related neurological disorders including macular degeneration and dementia [6,13]. Therefore, the supplemental consumption of polyphenolic antioxidants compounds by people could reduce the risk of AD.

Recently, plant extracts have been the subject of a lot of research in order to obtain compounds able to inhibit AChE. Most of these studies indicate that plants are a good source of molecules with this inhibition activity [14,15]. Most of the compounds isolated from the plant polar extract fraction are polyphenols [16,17]. These compounds also have a high antioxidant activity [16,18]. The antioxidant activity found in some compounds has been connected to the capacity to scavenge the free radicals that are formed during the inflammation processes [19].

As part of our studies on potential chemopreventive agents, we have evaluated the antibutyrylcholinesterasic, antiradical, and antioxidant effects of aqueous extracts from *Rhus pentaphyllum *collected from Melloulech in the center of Tunisia.

## 1. Materials and methods

### 1.1. Chemicals

1,1-diphenyl-2-picryl-hydrazyl (DPPH), allopurinol, α-tocopherol, nitroblue- tetrazolium (NBT), 6-hydroxy-2,5,7,8-tetramethylchroman-2-carboxylic acid (Trolox), 2,2'-azino-bis(3-ethylbenzothiazoline-6-sulfonic acid) diammonium salt (ABTS^**.+**^) were obtained from Sigma Co (St. Louis, USA). Butyrylthiocholine iodide and 5,5'-dithiobis [2-nitrobenzoic acid] (DTNB) were purchased from Quimica Clinica Aplicada S.A. (Amposta, Spain).

### 1.2. Plant materials

*R. pentaphyllum *was collected from station of Melloulech situated in the Center east of Tunisia in December 2008. Botanical identification was carried out by Dr. Amer Aissi (Pharmacognosy laboratory Faculty of Pharmacy Monastir - Tunisia). A voucher specimen (RP-10.03) has been deposited in the High Biotechnological Institute Sidi Thabet, for future reference.

### 1.3. Extraction Procedure

Three aqueous extracts were prepared from respectively the powdered leaves, root and seeds by boiling in water for 1 h. The extracts were filtered and lyophilized, and the residues were dissolved in water.

### 1.4. Preliminary phytochemical analysis

The various aqueous extracts were screened for the presence of tannins and flavonoids by using the methods previously described by Mansour *et al. *[20] Two milligrams of each extract were dissolved in 2 ml of water. The identification of major chemical groups was carried out by thin layer chromatography (TLC) on silica gel 60 F254 Merck (layer thickness, 0.25 mm), as follows. For flavonoids, the TLC was developed in n-butanol/acetic acid/water (4:1:5), and the spots were visualized with 1% aluminium chloride in methanol under UV (366 nm). The test for tannins was carried out with FeCl_3_. Each class of tannins produced a specific color.

### 1.5. Quantitative analysis of extracts

Flavonoids were quantified by using the method described by Dohou et-al [21]. Twenty milligrams of each extract were dissolved separately in 2 ml of 80% methanol and sonicated (30 sec, 100%) with a Sonics vibra-cell ultrasonic processor (Bioblock Scientific, Illkirch, France). After addition of 100 μl of diphenylborinic acid 2-aminoethyl ester (1% (w/v) in methanol) to each solution, the absorbance of flavonoids was determined spectrophotometrically at 404 nm and compared to a quercetin standard (0.05 mg/ml). The percentage of total flavonoids was then calculated in quercetin equivalents according to the following formula:

$$\text{F}=\left(0.05\;{\text{A}}_{\text{ext}}∕{\text{A}}_{\text{q}}\right)\;100∕{\text{C}}_{\text{ext}}$$

where Aext and Aq were the absorbance of the extract and of quercetin, respectively, and Cext was the extract concentration (10 mg/ml).

Tannins were quantified according to the method developed by Porter *et al. *[22] and adapted by Mansour *et al. *[20]. Solutions (1 g/l) of each extract were sonicated (30 sec, 100%), distributed in glass tubes, and sealed with a Teflon-lined screw cap. 2.5 ml of n-butanol-HCl (95:5, v/v) and 100 μl of a 2% (w/v) ferric reagent (NH_4_Fe (SO_4_)_2_. 12H_2_O) were added to each tube. The solutions were capped, thoroughly mixed, and suspended in a constant-level water bath at 95°C for 40 min. The solutions were cooled and the visible spectrum was determined at 540 nm. The percentage of total condensed tannins was then calculated in cyanidol (standard) equivalents according to the following formula:

$$\text{T}=\left[\left({\text{A}}_{\text{54}0\text{nm}}∕\in \text{l}\right)\text{1}∕{\text{C}}_{\text{ext}}\right]\;\text{1}00$$

Where l = 1 cm, Є = 42390 l/mol/cm, and C_ext _is extract concentration.

### 1.6. *In vitro *Butyrylcholinesterase inhibition assay

#### Human plasma preparation

Human blood from anonymous healthy men subject (27 years) was provided by the Centre d'Assistance Médical Urgente (C.A.M.U) Hôpital Charles Nicolle in Tunisia. Blood was collected in EDTA treated (1 mg/ml) glass tubes, the red blood cells were eliminated by centrifugation at 2000 g for 10 min, the plasma (supernatant) was then recuperated and diluted (1/200) with 50 mM phosphate buffer (pH = 7.4). Plasma was used immediately for studying butyrylcholinesterase (BuChE) activity or conserved at 2-8°C (stable for 7 days).

#### Butyrylcholinesterase inhibition assay

BuChE inhibiting activity was measured by the spectrophotometric method previously reported by Ellman *et al. *[23], modified by Ortega *et al. *[24] and adapted according to our experimental conditions. Butyrylthiocholine iodide was used as substrate to assay butyrylcholinesterase activity. In order to calculate the activity of the obtained butyrylcholinesterase, the following procedure was employed: 1.5 ml of phosphate buffer 50 mM pH = 7.2, containing 0.26 mM of 5,5'-dithiobis-2- nitrobenzoic acid (DTNB), 10 μl of human plasma and 10 μl of the tested compound (1, 10 and 100 μg/ml as final concentrations) were placed in a microcuvette, which was incubated for 15 min at 30°C. The hydrolysis of butyrylthiocholine was monitored by the formation of yellow 5-thio-2-nitrobenzoate anions resulting from the reaction of DTNB with the thiocholine released by the enzymatic hydrolysis of butyrylthiocholine. Absorbance was measured using an M350 double Beam UV-VIS spectrophotometer «Camespec» at 405 nm, and the reading was repeated during 75 s at intervals of 30 s to verify the linearity of the reaction. The enzymatic activity was calculated using the absorption coefficient 23460 and according to the relation:

$$\text{Enzymatic}\;\text{activity}\;\left(\text{UI}∕1\right)=23460\times \left(\text{D}{\text{O}}_{405\text{nm}}{\text{t}}_{0\text{s}}-\text{D}{\text{O}}_{405\text{nm}}\;{\text{t}}_{75\text{s}}\right)$$

The percentage (%) inhibition of BuChE activity was calculated as follows (E - S)/E × 100, where E is the activity of the enzyme without test compound (in our case E = 9 000 UI/l (international unite)) and S is the activity of enzyme with test compound.

IC_50 _(concentrations of test compounds that inhibited the hydrolysis of substrate (butyrylthiocholine) by 50%) values were calculated from dose-inhibition curves [25]. All experiments were repeated three times.

### 1.7. DPPH radical-scavenging activity

The free-radical scavenging capacity of the extracts was determined with DPPH [26]. Ethanol solutions were prepared containing 100, 30, 10, 3 and 1 μg/mL of the extracts and 23.6 μg/ml of DPPH. After incubation for 30 min at ambiant temperature, the absorbance of the remaining DPPH was determined colorimetrically at 517 nm. Radical scavenging activity was measured as the decrease in absorbance of the samples versus a DPPH standard solution [27]. Results were expressed as "percentage inhibition"(%) of the DPPH and the mean 50% inhibiting concentration (IC_50_). % is defined by the formula:

$$\left(\%\right)=\left[\left(\text{O}{\text{D}}_{\text{control}}-\text{O}{\text{D}}_{\text{sample}}\right)∕\text{O}{\text{D}}_{\text{control}}\right]\times 100,$$

Where OD_control _is the initial absorbance and OD_sample _the value for the test sample after incubation [27]. IC_50 _was defined as the concentration (in μg/ml) of substrate that causes 50% loss of DPPH activity (color) and it was calculated by using the Litchfield and Wilcoxon test [20].

The results are expressed as the mean of data from at least three independent experiments.

### 1.8. Radical-scavenging activity on ABTS^.+^

An improved ABTS^**.+ **^(2,2'-azino-bis (3-ethylbenzthiazoline-6-sulfonic acid) diammonium salt) radical cation decolorization assay was used [28]. It involves the direct production of the blue/green ABTS^+. ^chromophore through the reaction between ABTS^**.+ **^and potassium persulfate. Addition of antioxidants to the preformed radical cation reduces it to ABTS^**.+**^, to an extent and on a timescale depending on the antioxidant activity, the concentration of the antioxidant and the duration of the reaction [29]. ABTS^**.+ **^was dissolved in water to a 7 mM concentration. ABTS^+. ^was produced by reacting ABTS^**.+ **^stock solution with 2.45 mM potassium persulfate (final concentration) and allowing the mixture to stand in the dark at room temperature for 12-16 h before use. The ABTS^**+. **^solution was diluted with ethanol to an absorbance of 0.7 (± 0.02) at 734 nm. In order to measure the antioxidant activity of extracts, 10 μl of each sample at various concentrations (0.5, 2.5, 4.5, 7.5 and 9.5 mg/ml) was added to 990 μl of diluted ABTS^+• ^and the absorbance was recorded every 1 min. After 30 min the kinetic reaction was stopped. Each concentration was analyzed in triplicate. The percentage decrease of absorbance at 734 nm was calculated for each point and the antioxidant capacity of the test compounds was expressed as percent inhibition (%). IC_50 _value (concentration required to reduce ABTS^**+. **^by 50%) was calculated from regression analysis. Trolox (6-hydroxy-2,5,7,8-tetramethylchroman- 2-carboxylic acid) is used as a standard in comparison for the determination of the antioxidant activity of a compound. The results are also reported as the Trolox equivalent antioxidant capacity (TEAC), which is the molar concentration of the Trolox giving the same percentage decrease of absorbance of the ABTS^**+. **^radical cation as 1 mg/ml of the antioxidant testing extract, at a specific time point [29].

### 1.9. Superoxide radical-scavenging activity

The inhibition of NBT reduction by photochemically generated O_2_^.- ^was used to determine the superoxide anion scavenging activity of the extracts [30]. The reaction mixture contained 6.5 mM EDTA, 4 μM riboflavin, 96 μM NBT, and 51.5 mM potassium phosphate buffer (pH 7.4). Superoxide anions were measured by the increase in the absorbance at 560 nm after 6 min of illumination at room temperature. The plant extracts and the reference substance (Quercetin) were assayed at different concentrations with three repetitions. IC_50 _values (concentration required to inhibit NBT reduction by 50%) were calculated from dose-inhibition curves [31,20].

### 1.10. Statistical Analysis

Data were expressed as the mean 6 standard deviation of three independent experiments. The statistical analyses were performed with SPSS™ software v.10.0 (from SPSS Inc.). Data were analyzed for statistical significance using Dunnett's test.

## 2. Results

### 2.1. Phytochemical analysis

The results of our analysis on the lyophilized aqueous extracts are shown in table 1 and 2. Seeds aqueous extract contained the highest quantities of both flavonoids and tannins (21.12% and 17.45% respectively). The leaves extract had lower amounts of flavonoids and tannins (12.3% and 10.31%, respectively). Whereas, compared to the other extracts, the roots aqueous extract contained relatively high quantity of tannins, while flavonoids was not detected in this extract (table 2).

**Table 1:** Qualitative phytochemical screening of extracts from *Rhus pentaphyllum*

	Seeds aqueous extract	Leaves aqueous extract	Roots aqueous extract
Tannins	++	++	++++

Flavonoids	++++	++	-

Anthraquinones	-	-	-

Alkaloids	-	-	-

Coumarins	++	-	-

Saponosids	-	-	-

−: not detectable; ++: high quantities, ++++: very high quantities.

**Table 2:** Quantitative Phytochemical Screening (%) of extracts from *Rhus pentaphyllum *

	Seed aqueous extract	Leaves aqueous extract	Roots aqueous extract
Tannins	17.45*	10.31	35.51**

Flavonoids	21.12**	12.3**	0

Significant difference obtained with: *P < 0.05: **P < 0.01

The reported comparisons concern the contents of extracts in the flavonoids and tannins.

The qualitative phytochemical screening showed that only seeds extract contained coumarins (table 1).

### 2.2. *In vitro *butyrylcholinesterase inhibition effect

Results of human plasma BuChE inhibitory activity of the tested *R. pentaphyllum *extracts are shown in table IV. All tested extracts were found to inhibit the BuChE activity. The inhibition was instantly, as evidenced by the linearity of the absorbance *vs*. time traces during the 75 s assay period (*r^2 ^> 0.978*).

Results indicated that *R. pentaphyllum *extracts decreased significantly the human BuChE activity in a concentration-dependent manner (table 3).

**Table 3:** Percentage of inhibitions of butyrylcholinesterase activity by the three aqueous extracts from *Rhus pentaphyllum*

Tested compounds	Concentration (μg/ml)	Inhibition (%) against BuChE	IC_50_ (μg/ml)
Seeds aqueous extract	1	57.11 ± 2.00*	0.74
	10	80.34 ± 2.25**	
	100	94.93 ± 2.00**	

Leaves aqueous extract	1	54.78 ± 1.25*	0.81
	10	76.30 ± 1.50*	
	100	87.11 ± 5.50*	

Roots aqueous extract	1	32.25 ± 2.35	10.35
	10	49.01 ± 3.25	
	100	67.81 ± 3.67*	

^(a) ^Galanthamine	1	44.5 ± 1.00	7.9
	10	59.44 ± 2.5*	
	100	67.5 ± 2.5*	

Significant difference obtained with: *P < 0.05: **P < 0.01

The reported comparisons concern: Seeds aqueous extract versus control^(a)^, leaves aqueous extract versus control^(a) ^and roots aqueous extract versus control^(a)^. Every concentration is compared with its equivalent in the other extract.

Seeds and leaves aqueous extract displayed remarkable inhibition over 50% (95% and 87%, respectively) at 100 μg/ml against BuChE and with IC_50 _0.74 and 0.81 μg/ml. Roots aqueous extract have somewhat lower inhibitory activity with IC_50 _value 10.35 μg/ml.

### 2.3. Antioxidant activities

Oxidative effect of plant extracts cannot be evaluated by only a single method. Therefore, commonly accepted assays, including enzymatic and nonenzymatic methods, were employed to evaluate oxidative effects of some medicinal plants. Three different reactive species were used to evaluate the antioxidant activity of the *R. pentaphyllum *extracts; the ABTS^**.+**^, DPPH and superoxide radicals.

#### DPPH radical-scavenging activity

DPPH is a molecule containing a stable free radical. The presence of antioxidant substances could be revealed by the decrease of the intensity the purple color typical of the free DPPH radical [32]. This simple test can provide information on the ability of a compound to donate an electron, the number of electrons a given molecule can donate, and the mechanism of antioxidant action. The radical-scavenging activities of the extracts measured as decolorizing activity following the trapping of the unpaired electron of DPPH are shown in Table 4.

**Table 4:** DPPH free-radical scavenging activity of extracts from *Rhus pentaphyllum*

Extracts	Concentration (μg/ml)	% Inhibition	IC_50_ (μg/ml)
Seeds aqueous extract	1	44.34 ± 2.30*	2.71
	3	53.45 ± 1.02*	
	10	66.90 ± 1.10*	
	30	80.51 ± 2.80**	
	100	92.12 ± 2.11**	

Leaves aqueous extract	1	39.45 ± 0.91	2.91
	3	50.45 ± 0.75*	
	10	64.31 ± 1.50*	
	30	79.50 ± 2.80*	
	100	88.11 ± 2.55**	

Roots aqueous extract	1	12.12 ± 0.80	10.10
	3	34.51 ± 1.35	
	10	49.51 ± 2.10	
	30	63.67 ± 1.12*	
	100	70.11 ± 3.11*	

^(a) ^α-Tocopherol (positive control)	1	30 ± 2.1	3
	3	50 ± 1.3	
	10	97.3 ± 1.8**	
	30	98 ± 1.3**	
	100	98.7 ± 2.2**	

Significant difference obtained with: *P < 0.05: **P < 0.01,

The reported comparisons concern: Seeds aqueous extract versus roots aqueous extract, leaves aqueous extract versus roots aqueous extract and control^(a) ^versus roots aqueous extract. Every concentration is compared with its equivalent in the other extract.

The seeds and leaves aqueous extracts were very potent radical scavengers, with a percentage decrease versus the absorbance of the DPPH standard solution of 90 and 78%, respectively, at a concentration of 100 μg/ml, and IC_50 _values of 2.71 and 2.91 μg/ml. These values were slightly greater than that of the positive control, 3 μg/ml α-tocopherol. Aqueous extract (100 μg/ml) obtained from roots have scavenging activity of 70% and have IC_50 _value of 10.10 μg/ml.

#### Radical-Scavenging activity on *ABTS^.+^*

The free radical scavenging capacity of *R. pentaphyllum *extracts was evaluated by ABTS^**.+ **^assay (Table 5). Decolorization of ABTS^**.+ **^reflects the capacity of antioxidant species to donate electrons or hydrogen atoms to inactivate this radical cation. A potential activity was noted at different tested concentrations of all extracts studies (table 3). Tested extracts seem to be more actives than Trolox (reference), as IC_50 _value obtained with trolox (0.76) is greater than that obtained with the seeds, and leaves aqueous extracts (0.25, 0.37 mg/ml). Roots aqueous extract have somewhat lower inhibitory activity with IC_50 _value 2.31 mg/ml.

**Table 5:** Concentration-dependent ABTS^**.+ **^free radical scavenging activity of *Rhus pentaphyllum *aqueous extracts and standard antioxidant Trolox

Extracts	Concentration (mg/ml)	Inhibition (%)	TEAC (mM)	IC_50 _(mg/ml)
Seeds aqueous extract	0.5	76.4 ± 3.50*	2.19	0.25
	2.5	86.12 ± 4.25**		
	4.5	98.77 ± 6.50**		
	7.5	100 ± 1.00***		
	9.5	100 ± 1.50***		

Leaves aqueous extract	0.5	61.7 ± 3.50*	1.54	0.37
	2.5	87.8 ± 4.40**		
	4.5	89 ± 1.00**		
	7.5	98 ± 0.50**		
	9.5	100 ± 2.50***		

Roots aqueous extract	0.5	44.1 ± 0.35	1.32	2.31
	2.5	54 ± 1.10*		
	4.5	67.13 ± 3.10*		
	7.5	78.61 ± 5.10*		
	9.5	86.50 ± 2.35**		

^(a) ^Trolox	0.5	22.07 ± 0.25	-	0.76
	0.625	32.21 ± 0.50		
	0.833	53.84 ± 1.50		
	1.25	65 ± 2.50		
	2.5	96.85 ± 2.80**		

TEAC: Trolox equivalent antioxidant capacity

Significant difference obtained with: *P < 0.05; **P < 0.01; ***P < 0.001

The reported comparisons concern: Seeds aqueous extract versus roots aqueous extract, leaves aqueous extract versus roots aqueous extract and control^(a) ^versus roots aqueous extract. Every concentration is compared with its equivalent in the other extract.

The TEAC of different extracts was also calculated. The TEAC values reflect the relative ability of hydrogen or electron-donating antioxidant of a sample to scavenge the ABTS^**.+ **^radical cation compared with that of Trolox. The results obtained are summarized in table 5. When referring to TEAC values, seeds, leaves and roots extracts were potent antioxidant with TEAC values of respectively 2.19, 1.54 and 1.32 mM, which largely exceed 1 mM, the TEAC value of positive control (Trolox).

#### Effects on superoxide anion generating systems

**Figure 1:**
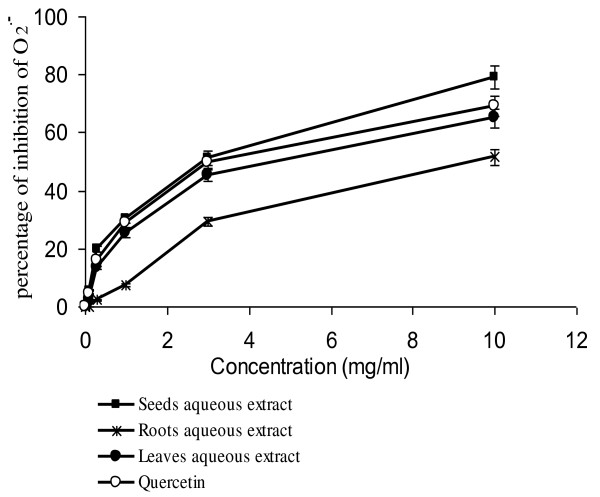
**Scavenging effects of aqueous extracts of *R. pentaphyllum *against photochemically generated superoxide free radicals (O^.-^_2 _)**.

The superoxide radical (O^.-^_2_) is a highly toxic species that is generated by numerous biological and photochemical reactions. Via the Haber-Weiss reaction, it can generate the hydroxyl radical, which reacts with DNA bases, amino acids, proteins, and polyunsaturated fatty acids, and produces toxic effects. The toxicity of the superoxide radical also could be due to the perhydroxyl intermediate (HO_2_) that reacts with polyunsaturated fatty acids. Finally, superoxide may react with NO to generate peroxynitrite, which is known to be toxic towards DNA, lipids, and proteins. The NBT assay is based on the capacity of the extracts to inhibit the photochemical reduction of nitroblue tetrazolium (NBT) in the presence of riboflavin. Under these conditions, NBT can be unevenly reduced in the presence of the O^.-^_2 _radical to a tetrazoinyl radical that can dismute to the formazan. In the presence of an antioxidant that can donate an electron to NBT, the purple color typical of the formazan decays, a change that can be followed spectrophotometrically at 560 nm. Results indicated that *R. pentaphyllum *extracts decreased significantly the NBT/riboflavin-generated superoxide radical in a concentration-dependent manner. Seeds aqueous extract seems to be more potent antioxidant with activity percentage of 79% at the highest concentration (10 mg/ml) compared to the other test extracts and an IC_50 _of 2.9 mg/ml. The seeds aqueous extract was more active than the positive control, quercetin, in the assay (Figure 1). The leaves and roots extracts had somewhat lower inhibitory activity with IC_50 _values of respectively 4.9 and 9.85 mg/ml.

## 3. Discussion

Principal role of cholinesterase (ChE) is the termination of nerve impulse transmission at the cholinergic synapses by rapid hydrolysis of acetylcholine (ACh). Inhibition of ChE serves as a strategy for the treatment of Alzheimer's disease (AD), senile dementia, ataxia, myasthenia gravis and Parkinson's disease [33,34] A variety of plants has been reported to show ChE inhibitory activity and so may be relevant to the treatment of neurodegenerative disorders such as AD [15].

In this study, aqueous extracts prepared from leaves, seeds and roots from *R. pentaphyllum *were tested to determine their ability as human BuChE inhibitors. The BuChE inhibition was determined using an adaptation of the method described by Ellman, *et al. *[23].

All extracts exhibited moderate to good anti BuChE activity, in fact, the inhibition capacity shows the following order: seeds extract > leaves extract > roots extracts. The best inhibitory activity was exhibited by the seeds extract.

On the other hand, the role of oxidative stress in the pathogenesis of diseases such as macular degeneration, certain types of cancer, and Alzheimer's disease (AD) has received substantial attention. For that reason, we also aimed to look into antioxidant capacities of *R. pentaphyllum *extracts.

Three different reactive species were used to evaluate the antioxidant activity of the *R. pentaphyllum *extracts: the DPPH^. ^, ABTS^**.+ **^and O_2_^.-^radicals. The superoxide anion and other ROS contribute to oxidative stress, and are known contributors to genetic damage, as well as degenerative diseases such as cancer [35], Parkinson disease, and heart ischemia [36]. Since, the DPPH^. ^And the ABTS^**.+ **^radicals are not biologically relevant, the DPPH and ABTS^**.+ **^assays were performed as a preliminary study to estimate the direct free-radical scavenging abilities of the test extracts. The activity of extracts against the superoxide radical via the non enzymatic NBT/riboflavin assay system has more relevance to physiological conditions. Results show that, compared to leaves and roots extracts, seeds aqueous extract revealed relatively strong antiradical activity towards the ABTS^**.+ **^and DPPH free radicals and decreased significantly the O_2_^.- ^formation. Thus, we can suggest that the anti-BuChE activities occurs through free radical scavenging capacities.

The antioxidant and anti-BuChE possibilities of *R. pentaphyllum *extracts are supported by the detection of flavonoids and phenolic compounds. In fact, several flavonoids and other phenolic compounds are considered antioxidants [37,20] and inhibition capacities of BuChE activity [38,15].

It has been reported, oxidative stress, caused by reactive oxygen species (ROS), is known to cause the oxidation of biomolecules leading to cellular damage. It is also speculated to be pathologically important in various neurodegenerative processes including cognitive deficits that occur during normal cerebral aging, Alzheimer's (AD), and Parkinson's diseases [39,40]. Nowadays, the most accepted theory about the disturbing effect of free radicals in the process of aging was reported by Harman [41]. Later on, it was also reported that oxidative stress is associated with the pathogenesis of AD and cellular characteristics of this disease are either causes or effects of oxidative stress [42,43]. These evidences clearly show that oxidative stress, an early event in AD, may play a key pathogenic role in the disease [44]. Thus, we can establish a correlation between the antioxidant and anti-BuChE capacities and quantity of these phenolic components. Curiously, the roots aqueous extract contained a high quantity of tannins but it exhibited lowest both antioxidant and anti-BuChE activities than the two other extracts. We cannot, however, exclude the possibility that other compounds, particularly coumarins in the case of seeds aqueous extract, with decreased the BuChE and free radical properties [45]. On the other hand, it is not necessarily always to be only one compound that is responsible for these effects, which may as well be depend on several compounds that act in a synergistic manner or on compounds which regulate one another.

In summary, *R. pentaphyllum *extracts appear to contain compounds with antioxidant and chemoprotective properties. Therefore, these data suggest that high dietary or supplemental consumption of antioxidants in people may reduce the risk of AD. However, further studies are required to fractionate the active extracts, to identify the active compounds, and to determine their exact mechanism of action.

## Competing interests

The authors declare that they have no competing interests.

## Authors' contributions

HBM is the primary author of the manuscript, planed the work, assisted in extracts preparation from powdered *R. pentaphyllum *roots, leaves and seeds and their chemical characterized. SY contributed in the antibutyrylcholinestrasic activity of all extracts. SM helped in the antioxidant activity against 1,1-diphenyl-2-picryl-hydrazyl (DPPH). AD participated in the antioxidant activity against 2,2'-azino-bis(3-ethylbenzothiazoline-6-sulfonic acid) diammonium salt (ABTS^**.+**^). IH participated in the antioxidant activity against superoxidae anion used by the non-enzymatic system nitroblue- tetrazolium (NBT). DD contributed in the statistical analyzes of data.

All the authors read and approved the final version of the manuscript.
